# Automated lesion tracking and treatment response classification on [^68^Ga]Ga-PSMA-11 PET/CT across PPP, RECIP 1.0 and TLW frameworks

**DOI:** 10.1007/s00259-026-08071-2

**Published:** 2026-07-16

**Authors:** Theo Lucas, Mikaela Dell’Oro, Jeremy S. L. Ong, Martin A. Ebert, Kaylee Molin, Jake Kendrick

**Affiliations:** 1 School of Physics, Mathematics and Computing, University of Western Australia, Perth, WA Australia; 2 Centre for Advanced Technologies in Cancer Research, Perth, WA Australia; 3 Curtin Medical School, Curtin University, Perth, WA Australia; 4 Department of Nuclear Medicine, Fiona Stanley Hospital, Murdoch, WA Australia; 5 Department of Radiation Oncology, Sir Charles Gairdner Hospital, Perth, WA Australia

**Keywords:** PSMA PET, Biochemically recurrent prostate cancer, Lesion-tracking, RECIP 1.0, PPP, TLW

## Abstract

**Purpose:**

This study aimed to validate an automated lesion-tracking algorithm for [^68^Ga]Ga-PSMA-11 PET/CT against expert reference matches and to evaluate its integration into three PSMA-specific treatment response frameworks—the Response Evaluation Criteria in PSMA PET/CT (RECIP 1.0), PSMA PET Progression (PPP) criteria, and the Traffic Light Workflow (TLW)—in men with biochemically recurrent (BCR) prostate cancer (PCa).

**Methods:**

Paired baseline and follow-up [^68^ Ga]Ga-PSMA-11 PET/CT scans from 132 BCR PCa patients were analysed using a registration-based pipeline that automatically assigned one-to-one lesion correspondences. Lesions were labelled as corresponding (CL), disappearing (DL) or new (NL). Algorithm-derived RECIP 1.0, PPP, and TLW categories were compared with expert reference labels and tested for overall survival (OS) association using univariable Cox regression.

**Results:**

A total of 1,066 lesions were identified (559 baseline, 507 follow-up), including 371 CLs, 188 DLs, and 136 NLs, yielding 695 matching decisions. The lesion matching algorithm achieved 95.1% matching accuracy. Lesion mismatches were associated with poorer registration quality. Algorithm-derived patient-level response classifications showed high concordance with ground-truth frameworks, with classification accuracy of 95.5% for RECIP 1.0, 96.2% for PPP, and 96.2% for TLW. All frameworks retained prognostic significance for OS, with progressive disease conferring hazard ratios (HR) of—RECIP 1.0 HR = 2.57 (95% CI 1.33–4.98), PPP HR = 2.02 (95% CI 1.04–3.90), and TLW HR = 2.29 (95% CI 1.22–4.28).

**Conclusion:**

The automated lesion-tracking algorithm reproduced expert matches with high accuracy and preserved the prognostic utility of RECIP 1.0, PPP, and TLW in a BCR cohort, supporting its use as a reproducible lesion-matching method for quantitative response assessment on PSMA PET/CT.

**Trial registration number:**

ACTRN ACTRN12615000608561. Registered 11 June 2015. Retrospectively registered.

**Supplementary Information:**

The online version contains supplementary material available at https://doi.org/10.1007/s00259-026-08071-2.

## Introduction

Prostate-specific membrane antigen (PSMA) is a transmembrane glycoprotein that is markedly overexpressed on the surface of most prostate cancer (PCa) cells [1]. PSMA-targeted positron-emission tomography/computed tomography (PET/CT) has demonstrated higher sensitivity and specificity than conventional imaging for detecting sites of PCa recurrence and metastasis [2, 3]. PSMA PET/CT enables highly specific, whole-body visualisation of PSMA-expressing disease and has subsequently transformed PCa staging, restaging, and treatment evaluation, frequently altering clinical management [4]. There is growing interest in using PSMA PET as a quantitative imaging biomarker to monitor therapy response and to stratify prognosis across different stages of PCa, including the biochemical-recurrent (BCR) stage, defined as rising serum prostate-specific antigen (PSA) levels following systemic therapy [5].

Despite the rapid adoption of PSMA PET, approaches to response assessment remain non-uniform, with centres variably applying qualitative reads, SUV-based thresholds, or volumetric criteria—hindering cross-study comparability and consistent clinical interpretation [6]. To address this gap, several PSMA-specific frameworks have been proposed, including the PSMA PET Progression (PPP) criteria, Response Evaluation Criteria in PSMA PET/CT (RECIP 1.0), and the newly proposed Traffic-Light Workflow (TLW) [7–9]. Because these frameworks rely on changes in individual lesions—such as the appearance of new lesions, resolution of existing ones, or variation in PSMA-avid tumour volume across persisting lesions—they inherently require accurate lesion-to-lesion correspondence between baseline and follow-up scans. Implementing this step clinically is challenging, as patient scans often contain numerous lesions exhibiting complex spatial and temporal evolution and manual matching is labour-intensive and prone to inter- and intra-observer variability, especially when lesion burden is high [10]. Automated lesion-tracking approaches can standardise this process and enable reproducible computation of framework inputs, including new-, disappearing-, and corresponding-lesion metrics.

Accordingly, the primary aims of this study were to (i) validate an existing automated lesion-tracking algorithm against expert reference matches on paired baseline/follow-up [^68^Ga]Ga-PSMA-11 PET/CT scans; (ii) apply its lesion-level outputs to generate patient-level classifications under three treatment evaluation frameworks (RECIP 1.0, PPP, TLW); (iii) further test whether algorithm-derived labels retained prognostic significance for overall survival (OS).

## Methods

### Patient cohort

This study comprised data for 238 men enrolled in a prospective trial (ACTRN12615000608561) at Sir Charles Gairdner or Fiona Stanley Hospitals (Perth, Western Australia). Ethics approval was granted by Sir Charles Gairdner HREC (RGS1736), Fiona Stanley HREC (2015–125), and The University of Western Australia (2019/RA/4/20/6382). The study was conducted in accordance with the principles outlined in the Declaration of Helsinki. Detailed inclusion criteria for the trial can be found in Molin et al. [11, 12]. Management strategies for the patients included active surveillance and any of the following therapies: additional surgery, radiotherapy to the prostatic bed or metastatic lesions, chemotherapy and/or androgen deprivation therapy. Eligibility for lesion tracking required paired baseline and follow-up scans with corresponding PET and CT at both timepoints and ≥ 1 lesion on either scan. Of the 238 patients, 200 had received a [^68^Ga]Ga-PSMA-11 PET/CT scan at both baseline and follow-up, approximately 6 months apart, following intervention. Of the 200 patients, 67 had no disease on either baseline or follow-up scan, and one patient had missing CT data. This left 132 patients to be considered for the present analysis.

### Scan acquisition and lesion delineation

[^68^Ga]Ga-PSMA-11 PET/CT scans were acquired on either a Siemens Biograph 64 or Biograph 128 PET/CT system (CTI Inc., Knoxville TN). Patients were instructed to void their bladder before imaging. Approximately 60 min after intravenous administration of 2 MBq/kg [^68^Ga]Ga-PSMA-11, a low-dose CT (120 kVp, 50 mAs) was performed for attenuation correction, followed immediately by PET emission over the same field of view. PET data were reconstructed to 4.07 × 4.07 mm^2^ pixels; CT data to 0.98 × 0.98 mm^2^ or 1.52 × 1.52 mm^2^ pixels, both with 2 mm slice thickness. Scanner performance was quantitatively harmonised under the Australasian Radiopharmaceutical Trials Network (ARTnet) accreditation scheme [13].

All patient scans were interpreted and segmented by an experienced nuclear medicine physician (J.O.) according to the E-PSMA 5-point scoring system [14], with lesion matching across timepoints also performed manually. A semi-automated delineation method was used, using an initial standardised uptake value (SUV) threshold of 3 followed by manual refinement to remove physiological uptake and include any additional lesions below the threshold. Segmentations were performed using MIM Encore software (MIM Software Inc., Cleveland, OH, USA).

### Lesion-tracking pipeline

The automated lesion-tracking algorithm, adapted from Santoro-Fernandes et al. [15], was designed to categorise each unique lesion between baseline (BL) and follow-up (FU) scans as a corresponding lesion (CL), disappearing lesion (DL), or new lesion (NL). In the first stage, whole-body rigid and deformable registrations were applied to align FU CT images to the corresponding BL CT images, enabling the superposition of the associated segmentation maps to a common coordinate system. Transformations were computed using a six-parameter rigid model followed by a cubic B-spline deformable registration implemented in the PlatiPy framework [16]. Registration used the mean-squared-error (MSE) similarity metric with multi-resolution optimisation. Nearest-neighbour interpolation was applied to propagate segmentation masks to the baseline geometry, preserving label integrity. Small lesions (< 5 voxels) were temporarily dilated prior to resampling to reduce the risk that very small structures would disappear during nearest-neighbour mask propagation and were subsequently eroded to restore their original physical extent.

To accommodate residual registration misalignments, a conformal morphological dilation was applied to each lesion using a Euclidean distance transform. An adaptive dilation radius was computed for each lesion as half of the minimum inter-lesion distance, capped at 10 mm [17]. Lesion correspondences were then established using a combinatorial linear assignment formulation solved by the Hungarian (Munkres) algorithm [15, 18]. For each potential baseline-follow-up lesion pair, a cost function quantified dissimilarity, and assignments were optimised under one-to-one constraints. The primary analysis used a fractional-overlap cost function, which normalised voxel intersection by the smaller lesion volume. Additional optimisation details, including fixed-radius sensitivity analysis and alternative cost-function formulations, are provided in the Supplementary Materials.

Algorithm performance was primarily assessed in terms of matching accuracy, quantifying the proportion of correctly identified lesion correspondences relative to the reference annotations. To characterise class-wise performance – that is, the algorithm’s ability to correctly identify CL, DL and NLs – one-vs-all confusion matrices for each lesion category were constructed—CL vs (NL ∪ DL), DL vs (CL ∪ NL), and NL vs (CL ∪ DL). In each matrix, the counts represent true positives (TP), false positives (FP), false negatives (FN), and true negatives (TN), which were subsequently used to compute category-specific precision and recall. The errors were also evaluated quantitatively. Each lesion-level correspondence was annotated with (i) registration quality indicators—normalised cross-correlation (NCC) and MSE, reflecting global alignment accuracy between baseline and follow-up CTs; (ii) lesion-specific attributes, namely lesion volume; (iii) patient-level lesion burden; and (iv) anatomical site. For continuous variables (NCC, MSE, lesion volume, and total lesion count), error rates were evaluated across quartiles with Wilson 95% confidence intervals and compared using Mann–Whitney U tests. For the categorical (anatomical site) variable, Fisher’s exact tests were used to assess whether mismatch proportions differed between regions, with false-discovery-rate (FDR) correction applied for multiple comparisons.

### Treatment response evaluation frameworks

The validated lesion-tracking outputs (CL, DL, NL) were then translated into patient-level response using three published treatment evaluation frameworks designed specifically for PSMA imaging. RECIP 1.0 combines changes in total PSMA-avid tumor volume (PSMA-VOL) with the presence of new lesions to assign patients into one of four response/progression categories [8]. PSMA-VOL was calculated in each timepoint’s native image space prior to any registration or resampling. In PPP, progression is defined as ≥ 2 new PSMA-positive distant lesions, or 1 new lesion with clinical/laboratory corroboration and confirmatory imaging/biopsy, or a 30% increase in lesion size/uptake on FU scans. In this study, to operationalise PPP consistently, progression was defined as either ≥ 1 new lesion or a ≥ 30% increase in volume among CLs [7, 12]. Finally, the recently proposed TLW, which was introduced specifically in the context of [^1^⁷⁷Lu]Lu-PSMA therapy cohorts, first classifies lesions according to quantitative changes in volume or PSMA SUV_max_. Lesions are labelled green for a ≥ 30% volume decrease or a disappearing lesion, yellow for < 30% volume/PSMA SUV_max_ change, and red for a ≥ 30% volume/PSMA SUV_max_ increase or the appearance of a new lesion. At the patient level, TLW then aggregates these lesion-specific colour states to categorise overall response as responder, stable disease, low-volume progressor, or high-volume progressor, based on the predominance of the three colour states [9]. Table 1 summarises these three frameworks.

**Table 1:** Comparison of treatment response evaluation frameworks for PSMA PET/CT. RECIP 1.0 and PPP define patient-level progression primarily based on volumetric change and the appearance of new lesions. In contrast, TLW first performs a lesion-level classification and subsequently aggregates these lesion-level states to derive a patient-level classification. The LVP category represents a case where < 50% of the total post-treatment PSMA-VOL is dominated by increasing/new lesions, whereas the HVP category represents a case where ≥ 50% of total post-treatment PSMA-VOL is dominated by increasing/new lesions

RECIP 1.0	PPP	TLW	
Complete response (CR): Absence of any PSMA uptake on follow-up PET/CT	**Progression:** Appearance of ≥ 1 new PSMA-positive lesion or ≥ 30% increase in lesion volume between baseline and follow-up	**Lesion-level classification**	***Reducing**** (green):* ≥ 30% decrease in volume or complete resolution ***Stable**** (yellow):* < 30% change in volume or SUV_max_ ***Increasing**** (red):* ≥ 30% increase in volume or SUV_max_ ***New**** (red):* Lesion present only on follow-up
Partial response (PR): > 30% decrease in PSMA-VOL without new lesions			
Stable disease (SD): < 30% decrease or < 20% increase in PSMA-VOL with or without new lesions	**Non-progressive:** Do not exhibit progression	**Patient-level classification**	***Responder****:* No increasing/new lesions ***Stable disease****:* No reducing/increasing/new lesions ***Low- volume progressor (LVP)****:* < 50% increasing/new ***High-volume progressor (HVP):*** ≥ 50% increasing/new
Partial disease (PD): > 20% increase in PSMA-VOL and appearance of new lesions			

### Statistical analysis

Overall survival (OS) was defined as the interval from baseline PSMA PET/CT imaging to death, with censoring at the date of last follow-up for patients still alive. Associations between OS and treatment response classifications (RECIP 1.0, PPP, TLW) were evaluated using univariable Cox proportional hazards regression. Hazard ratios (HRs) and 95% confidence intervals (CIs) were estimated for both algorithmic and ground-truth classifications under each framework. All tests were two-sided, with *p* < 0.05 considered statistically significant. Analyses were performed in Python (lifelines v0.28.0).

## Results

### Patient characteristics

Across the 132 patients considered for the lesion-tracking analysis, a total of 1,066 individual lesions were identified, comprising 559 baseline and 507 follow-up lesions. Of these, 371 were classified as CL, 188 as DL, and 136 as NL, yielding 695 unique reference matching decisions.

The number of lesions per patient at baseline ranged from 1 to 34 (media*n* = 2; IQR: 1–4), while at follow-up, the number of lesions ranged from 1 to 55 (media*n* = 2; IQR: 1–4). Disease was predominantly nodal (69.2%, 481/695), followed by skeletal (18.6%, 129/695), prostate bed (7.2%, 50/695), non-skeletal/other (3.9%, 27/695), and prostate (1.2%, 8/695) involvement. Further detailed patient-specific characteristics are summarised in Table 2.

**Table 2:** Patient characteristics. Continuous data is presented as the median and the range, and categorical data is presented as the number and percentage

Characteristic	All patients (*n* = 132)
Age (y)	70.7 (45.8–90.0)
Weight (kg)	90.0 (60.0–128.0)
PSA (ng/mL)	4.4 (0.2–79.5)
Gleason Score*	
< 8 ≥ 8	68 (51.5%) 60 (45.5%)
Prior definitive treatment	
Prostatectomy Radiotherapy	63 (47.7%) 69 (52.3%)
Administered treatment	
Active surveillance ADT Radiotherapy Chemotherapy Surgery	20 (15.2%) 87 (65.9%) 40 (30.3%) 7 (5.3%) 3 (2.3%)

*Gleason score was missing for 4 patients

### Lesion-tracking performance

**Fig. 1:**
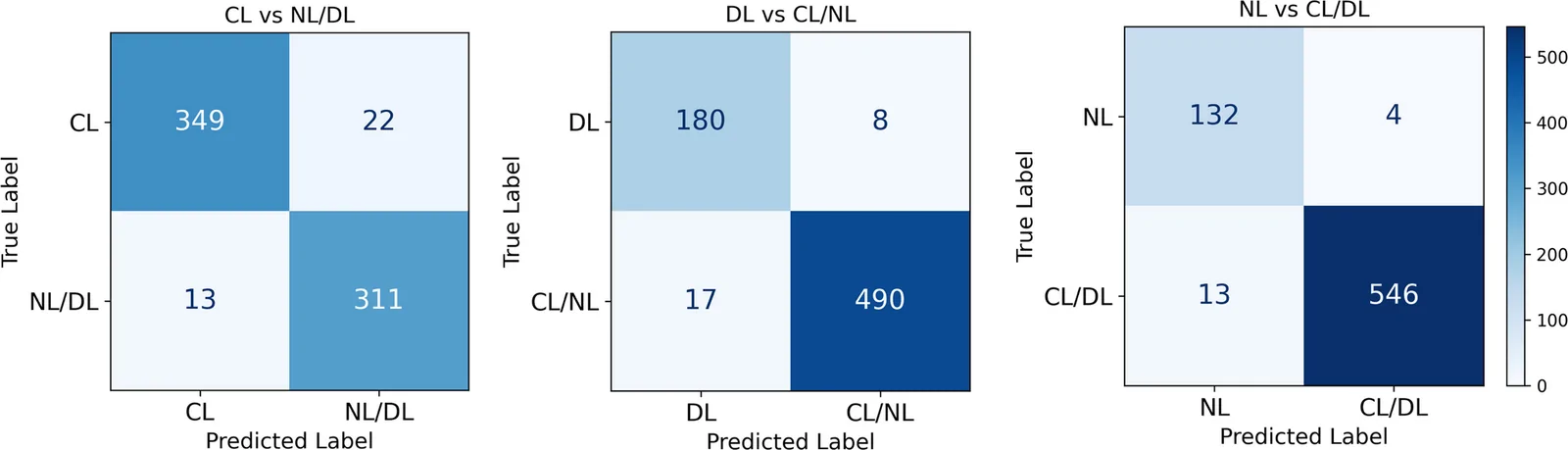
Lesion-level confusion matrixes showing TP, TN, FP and FN across the 3 lesion categories

Under the automatic-dilation framework, the best-performing cost formulation – an overlap-normalised cost that scales the voxel intersection by the smaller of the two lesion volumes (i.e., fractional overlap)—achieved 95.1% matching accuracy (661/695 decisions). Given its performance, this formulation was used for all subsequent analyses. Details of alternative cost-function formulations and their comparative performance are provided in the Supplementary Materials (Supplementary Table 2). Figure 1 displays the one-vs-all confusion matrices for the three lesion categories (CL, DL, NL), summarising the distribution of TP, FP, FN, and TN used to derive precision and recall.

**Fig. 2:**
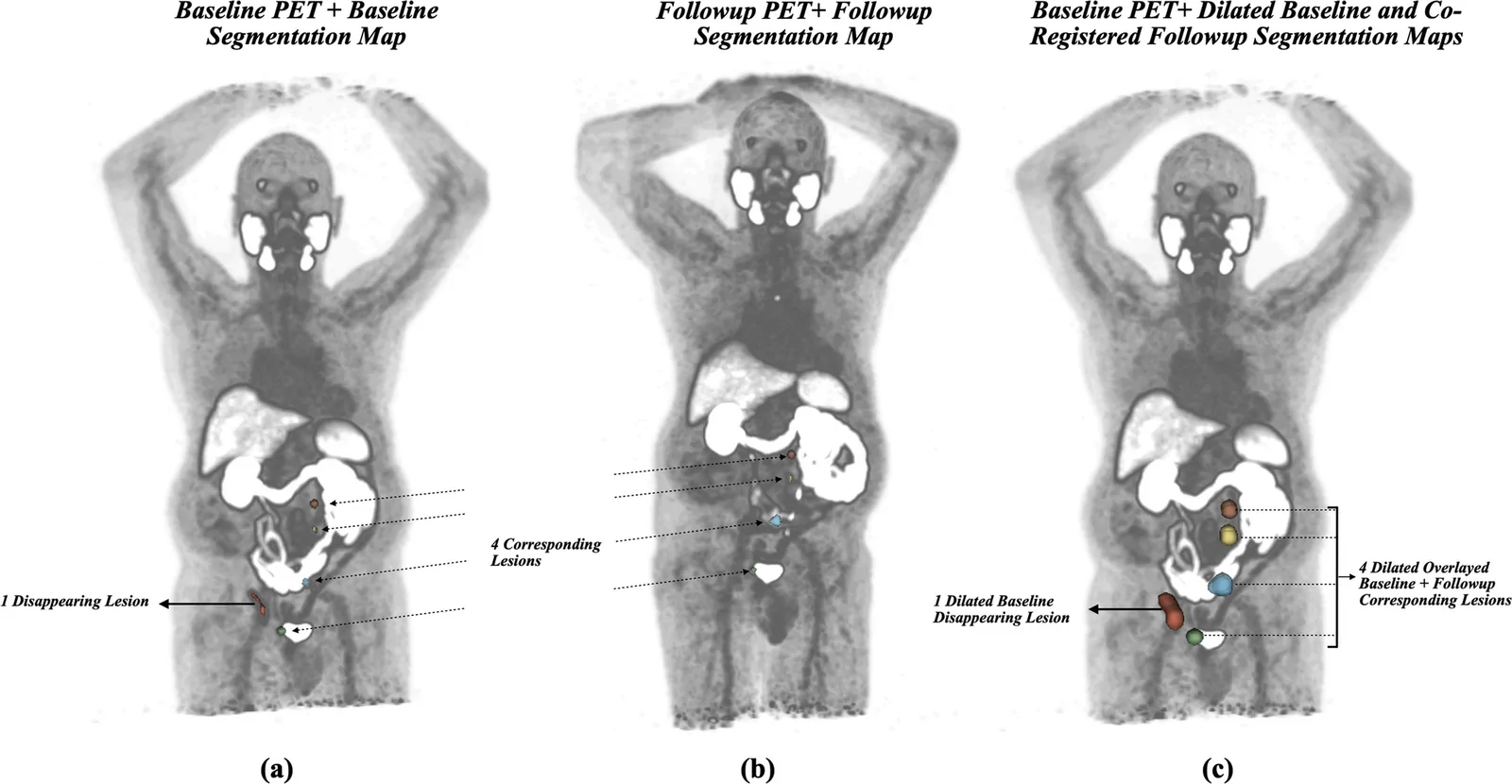
An example of a patient in whom the lesion-tracking algorithm performed successfully. This patient had 4 corresponding lesions (CL) between baseline (**a**) and follow-up (**b**) PSMA PET scans in the pelvic region. As a result of accurate registration during the lesion tracking workflow (NCC = 0.976, MSE = 4638), the mapped follow-up lesions aligned closely with their baseline counterparts, preserving spatial position and morphology in the common baseline coordinate system. Morphological dilation was then applied to both baseline and co-registered follow-up segmentations to accommodate residual registration uncertainty, as shown by the overlaid segmentation maps in panel (**c**). The degree of spatial overlap between dilated lesion pairs was subsequently maximised to determine lesion correspondence, and the high overlap achieved between each baseline–follow-up lesion pair led the algorithm to correctly identify the 4 CLs and 1 DL

The results showed the highest recall for detecting new lesions (97%), indicating that the algorithm rarely missed emerging disease, and highest precision for corresponding lesions (96%), confirming consistent one-to-one matching for stable or persisting disease. For disappearing lesions, the algorithm achieved precision of 91% and recall of 96%. Figure 2 presents an example of a patient in whom the lesion-tracking algorithm performed successfully, illustrating accurate lesion correspondence following registration.

Registration quality was strongly associated with the 34 mismatch decisions made by the algorithm. The lowest NCC quartile (0.756–0.967) exhibited an error rate of 10.8% (20/185; 95% CI 7.1–16.1%), compared with 1.2–3.7% in the higher quartiles. Similarly, for MSE, the highest quartile (> 7 600) showed 9.9% error rate (17/172; 6.3–15.3%) versus 2.5–4.7% elsewhere. These effects were statistically significant when comparing the lowest versus highest three quartiles (both *p* < 0.005), confirming that poorer registration—lower NCC or higher MSE—was strongly associated with mismatches, increasing risk by approximately two-to ten-fold across the observed range. Lesion size exerted a weaker, non-monotonic influence: smaller lesions (< 0.36 mL) had a higher error rate (5.6%), and larger lesions (> 1.59 mL) showed a modest rise (6.1%). However, when the full lesion-volume distributions were compared between mismatched and correct decisions, they were statistically indistinguishable (mismatched: median 979 mm^3^; correct: median 896 mm^3^; Mann–Whitney U, *p* = 0.96), suggesting that lesion size alone was not a key driver of mismatch errors. Similarly, the distribution of patient-level total lesion burden did not differ significantly between mismatched and correct decisions (mismatched: median 20.5 lesions; correct: median 15.0 lesions; Mann–Whitney U, *p* = 0.39). Consistent with this, mismatch rates remained between 3 and 6% across lesion-burden quartiles. These results are shown in Table 3.

**Table 3:** Quartile-level lesion matching error rates (with Wilson 95% confidence intervals) across key continuous variables. Quartiles were defined at the lesion-decision level. For lesion volume, each lesion-matching decision contributed its own value, whereas for NCC, MSE, and total lesion count, the corresponding patient-level value was assigned to each lesion-matching decision. Poorer registration quality (lower NCC, higher MSE) markedly increased mismatch risk, while lesion size and total lesion count showed only mild trends

Stratum	Quartile Range	Errors	Total	Error Rate (%)	95%CI_lo_	95%CI_hi_
*Registration Quality Metrics*						
NCC Q1	(0.756, 0.967]	20	185	10.8	7.1	16.1
NCC Q2	(0.967, 0.972]	6	163	3.7	1.7	8.0
NCC Q3	(0.972, 0.976]	2	173	1.2	0.3	4.1
NCC Q4	(0.976, 0.986]	6	174	3.5	1.6	7.3
MSE Q1	(2807.9, 4927.1]	5	192	2.6	1.1	6.0
MSE Q2	(4927.1, 5863.9]	4	160	2.5	1.0	6.3
MSE Q3	(5863.9, 7595.0]	8	171	4.7	2.4	9.0
MSE Q4	(7595.0, 48834.7]	17	172	9.9	6.3	15.3
*Lesion Size (Volume in mm*^*3*^*)*						
Q1	(33.2, 364.9]	10	179	5.6	3.1	10.0
Q2	(364.9, 895.7]	6	179	3.4	1.5	7.1
Q3	(895.7, 1592.4]	8	172	4.7	2.4	9.0
Q4	(1592.4, 122087.3]	10	165	6.1	3.3	10.8
*Total Lesion Count per Patient*						
Q1	(2, 7]	10	191	5.2	2.9	9.4
Q2	(7, 15]	5	159	3.2	1.4	7.1
Q3	(15, 30]	10	183	5.5	3.0	9.8
Q4	(30, 66]	9	162	5.6	3.0	10.2

By anatomical site, 28/34 errors occurred in nodal regions, consistent with the overall lesion distribution rather than indicating a site-specific bias. Although the nodal region had the highest absolute number of mismatches, the Fisher’s exact test showed no statistically significant differences in error rates between anatomical sites after FDR correction (*p* = 0.30).

### RECIP 1.0 and PPP classifications

Of the 132 patients, 31 (23.5%) had at least one new lesion on follow-up scans. The mean PSMA-VOL was 7.04 mL at baseline and 6.91 mL at follow-up (median 3.12 mL vs 2.07 mL), corresponding to a median percentage change of –20.3% (IQR –54.0% to 45.5%) from baseline to follow-up. The ground-truth RECIP 1.0 classifications were partial response (PR) = 47 (35.6%), stable disease (SD) = 66 (50.0%), and progressive disease (PD) = 19 (14.4%).

**Fig. 3:**
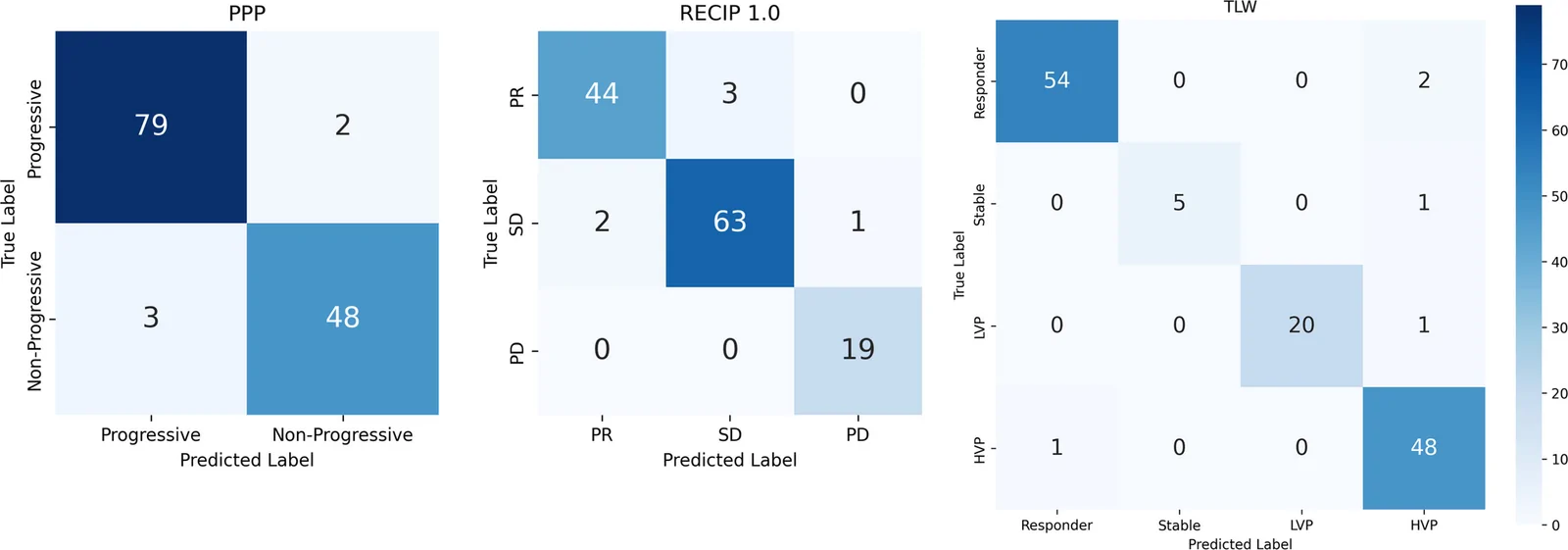
Patient-level confusion matrices comparing algorithmic versus ground-truth classifications across the three PSMA-PET response frameworks. Correct classifications are concentrated along the diagonal, indicating high concordance across all frameworks (overall accuracy: PP*P* = 96.2%, RECI*P* = 95.5%, TLW = 96.2%). Misclassifications were almost exclusively driven by new-lesion detection discrepancies rather than errors in corresponding-lesion volume change

At the patient level, the lesion-tracking algorithm correctly identified the presence or absence of new lesions in nearly all cases, leading to 126/132 (95.5%) correct RECIP 1.0 classifications under the automatic-dilation setting. Recall by category was 93.6% for PR (44/47), 95.5% for SD (63/66), and 100% for PD (19/19). The six misclassifications comprised three PR mislabelled as SD, two SD mislabelled as PR, and one SD mislabelled as PD. Figure 3 shows confusion matrices comparing algorithmic versus ground-truth classifications across RECIP 1.0, PPP, and TLW.

Across the 371 CL pairs, 128 (34.5%) showed ≥ 30% PSMA-VOL increase, 117 (31.5%) showed ≤ 30% decrease, and 126 (34.0%) remained relatively stable (− 30% to + 30% volume change). The lesion-wise volume percentage change had a median of − 3.7% (IQR − 54.7% to + 87.5%). Aggregated to the patient level, 66 patients had at least one lesion with ≥ 30% increase in volume, 71 had ≥ 1 lesion with ≤ 30% decrease, and 31 had both enlarging and shrinking lesions. Subsequently applying the PPP rule (a patient is progressive if presenting with a NL or ≥ 30% CL increase) gave the following ground-truth distribution: progressive 81/132 (61.4%), non-progressive 51/132 (38.6%). Across the 81 progressive patients, 50 had a > 30% increase in CL size, 15 had new lesions and 16 had both. The algorithm yielded a closely matched distribution corresponding to an overall classification accuracy of 96.2% (127/132). Precision for the progressive category was 96.3% and recall was 97.5%. All five disagreements were attributable to the new-lesion algorithmic mismatches (three FP-new; two FN-new); none arose from the ≥ 30% CL-increase arm. Thus, PPP disagreement reflected new-lesion detection errors rather than errors in corresponding-lesion volume changes.

### TLW classification

Across all CLs, median SUV_max_ declined from 6.18 at baseline to 4.71 at follow-up, with a median change of − 17.9% (IQR − 53.4% to + 28.1%). Mapping the lesions to the TLW categories yielded: reducing (green) = 399, stable (yellow) = 52, increasing (red) = 108, and new (red) = 136. Within the CL subset, lesions spanned all dynamic states (reducing = 211, stable = 52, increasing = 108). At the patient level (*n* = 132), the ground-truth TLW distribution was responder (*n* = 56), stable disease (n = 6), low-volume progressor (LVP, n = 21), and high-volume progressor (HVP, *n* = 49). As expected, the increasing-share metric (fraction of post-treatment PSMA-VOL that is “increasing” or “new”) clearly differentiated the progression categories: LVP patients had a median increasing-share of 0.197 (IQR 0.160–0.400), while HVP patients had a median of 1.00 (IQR 0.784–1.00). With the use of the lesion-tracking algorithm, 55 patients were labelled as responders, 5 as stable disease, 20 as LVP and 52 as HVP. There were thus 5 misclassifications, yielding an overall classification accuracy of 96.2%.

**Fig. 4:**
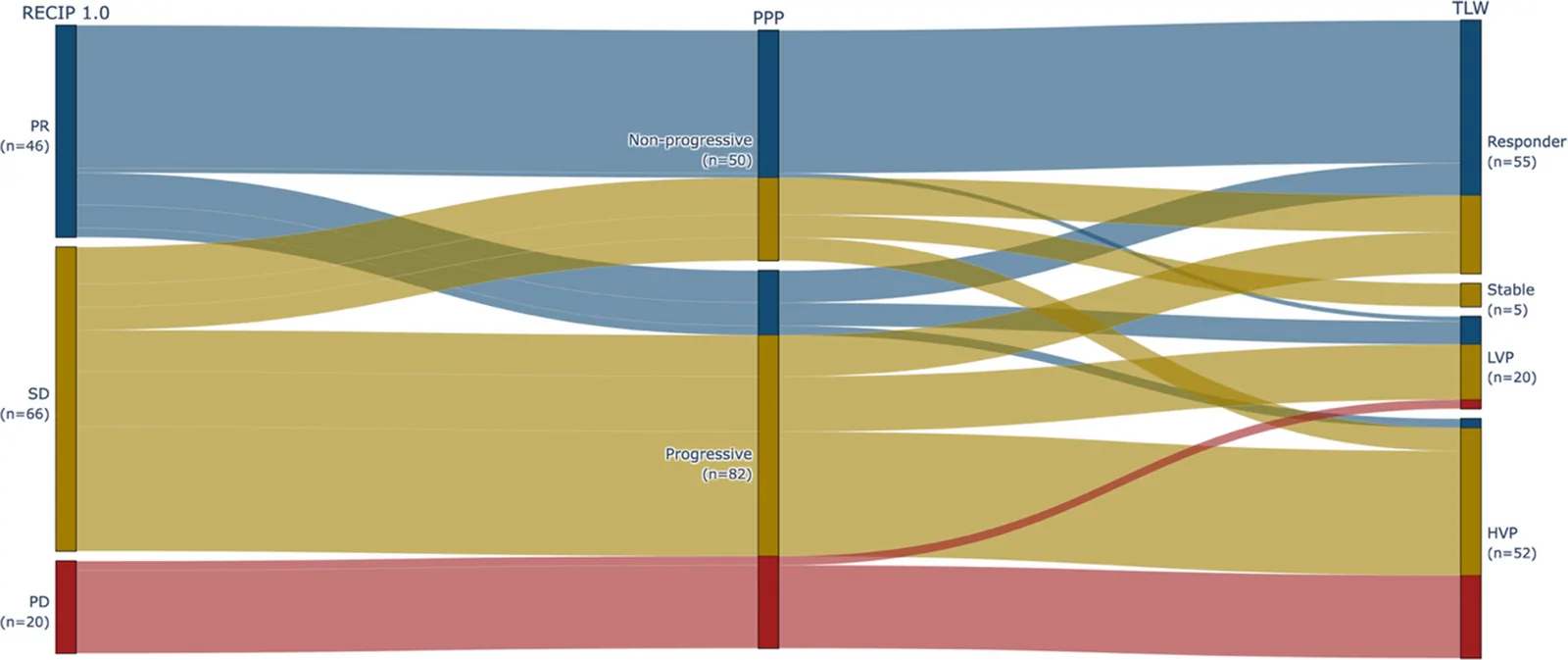
Parallel-categories diagram illustrating patient-level classifications across the three PSMA-PET response frameworks using the algorithm’s lesion correspondences. Each band represents a group of unique patients, with flows connecting their assigned categories under RECIP 1.0, PPP and TLW. While broad concordance was observed across the frameworks, several patients exhibited discordant classifications: for example, 14 patients labelled as partial response (PR) under RECIP 1.0 were deemed progressive under PPP and 2 of these patients were classified as high-volume progressors (HVP) under TLW. This highlights how variations in response criteria can shift patient-level classification

Among the five TLW patient-level misclassifications, four were driven by the NL pathway. Three patients had false-positive NL cases, meaning the algorithm incorrectly introduced additional new lesions. Each of these spurious NL contributed new voxel volumes to the post-treatment PSMA-VOL and was automatically counted toward the “increasing” compartment, thereby expanding the apparent tumour burden and pushing the increasing-share towards 1.00. This inflation triggered a categorical shift from responder or stable to HVP, since the TLW decision rule defines HVP when nearly all post-treatment disease volume is increasing or new. Conversely, one patient represented the opposite pattern—a false-negative NL—where omission of a new lesion led to an underestimated PSMA-VOL and a reduction of the increasing-share towards 0.00, causing a HVP to responder categorical reversal. The remaining misclassification reflected a correspondence-driven shift rather than new-lesion error: redistribution of volume among CL/DL pairs increased the algorithmic increasing-share from 0.102 to 0.888, resulting in a LVP to HVP re-categorisation. Collectively, these patterns indicate that TLW disagreement was largely dominated by the incorrect detection of NLs. Figure 4 illustrates how patients were categorised across the three response frameworks—RECIP 1.0, PPP, and TLW—with the use of the lesion-tracking algorithm.

**Fig. 5:**
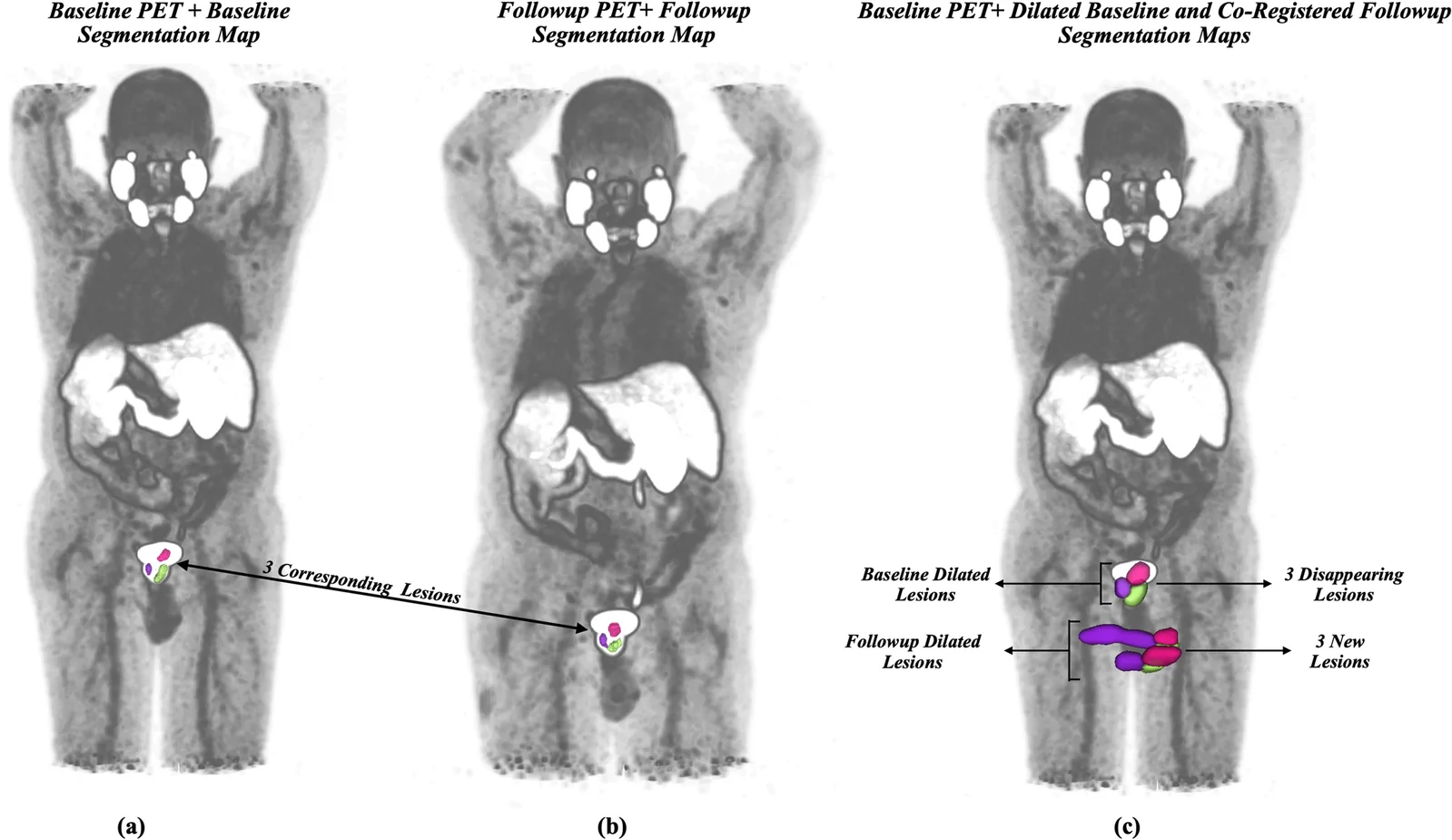
An example of a patient-level misclassification across RECIP 1.0, PPP, and TLW frameworks. This patient had 3 corresponding lesions (CL) between baseline (**a**) and follow-up (**b**) PSMA PET scans in the prostate bed. As a result of extremely poor registration during the lesion tracking workflow (NCC = 0.764, MSE = 40,841), and despite application of morphological dilation to compensate for registration uncertainty, the mapped follow-up lesions were warped inferiorly relative to the baseline prostate and distorted in shape in the common baseline coordinate system, as shown in panel (**c**). This loss of spatial overlap led the algorithm to incorrectly classify the lesions as 3 disappearing lesions (DL) and 3 new lesions (NL), rather than 3 corresponding lesions. This false-positive NL pattern caused the patient to be misclassified from PR → SD under RECIP 1.0, Non-progressive → Progressive under PPP, and Responder → High-volume progressor under TLW

Altogether, 8 unique patients were misclassified across the 3 frameworks. Figure 5 presents an example case. Given the lesion-level failure mode analysis identified registration metrics (NCC and MSE) as a dominant factor in mismatch errors, these metrics were compared between the misclassified (n = 8) and correctly classified patients (*n* = 124). For NCC, the misclassified group exhibited significantly lower values (mea*n* = 0.90 ± 0.09) compared with the remainder of the cohort (mea*n* = 0.97 ± 0.01, *p* = 0.002). Conversely, the MSE was much higher in misclassified patients (mea*n* = 21 611 ± 16 080) relative to others (mea*n* = 6 783 ± 2 942, *p* < 0.001). Collectively, these results demonstrate a clear association between degraded registration quality and patient-level misclassification.

### Survival analysis

Across the cohort, a total of 46 deaths (34.8%) and 86 censored observations (65.2%) were recorded. The median OS was 7.09 years (IQR 5.43–7.53 years). Univariable Cox models demonstrated that lesion‐tracking–derived classifications preserved prognostic relevance for OS, as shown in Table 4. The RECIP 1.0 framework yielded the strongest associations, with progressive disease corresponding to a significantly increased hazard of death (algorithm HR = 2.57, 95% CI 1.33–4.98; ground truth HR = 2.48, 95% CI 1.26–4.90). PPP classifications showed a consistent prognostic trend also, reaching significance for the algorithmic labels (HR = 2.02, 95% CI 1.04–3.90) but only a near‐significant trend for ground truth (HR = 1.83, 95% CI 0.96–3.47). For TLW, both ground-truth and algorithmic categorisations were prognostic (ground truth HR = 1.90, 95% CI 1.04–3.49; algorithm HR = 2.29, 95% CI 1.22–4.28), indicating that patients classified as progressive (HVP + LVP) had approximately double the risk of death compared with those with non-progressive disease (responder + stable). Comparison of C-index values showed only modest differences in prognostic discrimination across frameworks, with algorithm-derived TLW performing highest (0.60), followed by PPP (0.58) and RECIP 1.0 (0.57).

**Table 4:** Univariable Cox proportional hazards analysis for overall survival (OS) using patient-level classifications derived from RECIP 1.0, PPP, and TLW frameworks

Framework	Source	HR (95% CI)	*p*	C-index
PPP	Ground-truth	1.825 [0.961, 3.469]	0.066	0.57
	Algorithm	2.016 [1.044, 3.895]	0.037	0.58
RECIP 1.0	Ground-truth	2.480 [1.255, 4.898]	0.009	0.57
	Algorithm	2.571 [1.327, 4.981]	0.005	0.57
TLW	Ground-truth	1.900 [1.036, 3.487]	0.038	0.58
	Algorithm	2.285 [1.219, 4.284]	0.010	0.60

## Discussion

This study evaluated the performance of a registration-based lesion-tracking algorithm and assessed the feasibility of using the algorithmic-generated lesion correspondences in the context of three PSMA-specific treatment response evaluation frameworks: PPP, RECIP 1.0 and TLW. To our knowledge, although automated or AI-assisted longitudinal lesion tracking on PSMA PET/CT has been proposed previously, this is the first study to systematically embed lesion-tracking information into multiple PSMA response criteria and test them for preservation of OS prognostic power. Clinically, this approach could enable fully automated response assessments that are agnostic to how individual segmentations were generated, substantially reducing the manual workload of lesion matching and facilitating the rapid and reproducible stratification of patients for therapeutic decision-making.

Addressing the first study objective, the algorithm—originally introduced by Santoro Fernandes et al. [15]—reproduced expert lesion correspondences with a matching accuracy of 95.1%, a result comparable to, or better than, other published algorithms’ performances. Jönsson et al. had achieved a precision/sensitivity for matching lesions of 93%/87% for metastatic breast-cancer in FDG PET/CT scans using a registration-based approach, with performance dropping for lesions < 1 mL [19], an effect likely attributable to partial-volume and resampling effects during the registration process. This finding is consistent with our observation that smaller lesions (< 0.36 mL) tended to have a higher chance of producing mismatch errors; however, comparison of lesion sizes between correctly matched and mismatched lesions showed no statistically significant difference (Mann–Whitney U test, *p* = 0.92). Notably, our methodology differs from Jönsson et al.’s in two main aspects: a lesion-dilation step is incorporated, and they employ organ-segmentation masks to guide registration while the current method does not. Cai et al.’s Deep Lesion Tracker (a Siamese network trained on the NIH DeepLesion dataset — a public, multi-organ CT lesion collection [20]) reached 89% matching accuracy on 480 lesion pairs [21], while Hein et al. later applied a Siamese-CNN to PSMA PET/CT and obtained 83% correct matches across 1,490 reference matching decisions [22]. Graph-based formulations such as that of Di Veroli et al. showed excellent results in organ-specific regions (accuracies of 98% in lung and 85% in liver) but this was not designed for whole-body scenarios [23]. Santoro-Fernandes et al. had achieved a 98% matching accuracy from 736 matching decisions from [^18^F]FDG and [^18^F]FLT PET/CT scans [15]. This performance was obtained using a fixed 25 mm dilation radius. In our implementation, increasing the dilation radius progressively from 0 to 18 mm (see Supplementary Fig. 1) was found to decrease overall matching accuracy, with a maximum of 95.5% at 9 mm and a decline thereafter. Excessive dilation can begin to merge anatomically distinct lesions thereby complicating the optimisation of fractional overlap between lesion pairs, possibly leading to this decline. The current methodology instead adopted the adaptive, lesion-specific dilation strategy later proposed by the same group [17], which is likely to be more generalisable to other datasets, as the dilation radius dynamically scales with various inter-lesion spacings.

Addressing the second study objective, the translation of algorithm-derived lesion correspondences into patient-level treatment-response assessment represents an emerging paradigm in quantitative PSMA PET/CT analysis. For example, Kendrick et al. evaluated the prognostic utility of RECIP 1.0 in BCR PCa using both manual and AI-based [^68^Ga]Ga-PSMA-11 PET segmentations [24]. Their approach demonstrated that fully automated tumour segmentation preserved RECIP prognostic discrimination; however, the identification of new lesions remained a manual step, preventing complete automation of the response workflow. More recently, the updated aPROMISE platform, which can segment, measure, and temporally track PSMA-avid lesions, was used to assign RECIP 1.0 categories to 67 patients [25]. Five misclassifications occurred due to the platform falsely identifying new lesions, which led to five PR to SD re-categorisations. In comparison, the present automated lesion-tracking framework yielded six misclassified cases out of 132 patients—three of which were PR-to- SD changes- corresponding to a higher overall classification accuracy (95.5% vs 92.5%) and demonstrating that lesion-level outputs can be translated into highly concordant patient-level RECIP 1.0 classifications. Our work further demonstrated that algorithmic-derived RECIP 1.0 classifications retained prognostic ability, with RECIP-PD patients exhibiting a significantly higher risk of death (HR = 2.57, 95% CI 1.33–4.98, *p* = 0.005). Kendrick et al. reported a HR of 3.75 (95% CI: 1.23–11.47, *p* = 0.02) in 199 patients using AI-based segmentations and Molin et al. reported a higher HR of 4.62 (95% CI: 2.41–8.87; p < 0.001) in 154 patients, using semi-automatic segmentations with manual lesion correspondence identification, respectively, both derived from the same prospective cohort analysed in the present study [12, 24]. It should be noted, however, that our analysis used a smaller subset (*n* = 132) of the original prospective dataset and included 20 patients managed with active surveillance (AS), as they exhibited trackable lesions across timepoints, which likely attenuated hazard estimates — since AS is not a systemic treatment and RECIP 1.0 categorisation is designed for evaluating post-therapy response.

The PPP framework likewise demonstrated that the algorithm-derived progressive category preserved a prognostic trend (HR = 2.02, 95% CI 1.04–3.90, *p* = 0.037), an effect comparable to Molin et al.’s findings (HR = 2.88, 95% CI 1.55–5.35 < 0.001) [12]. Unlike RECIP 1.0, which is dependent upon the accurate detection of NLs, PPP incorporates both CLs and NLs into its progression logic. Despite this compounded dependence on both lesion correspondence, PPP achieved a slightly higher overall classification accuracy (96.2%) than RECIP 1.0, and all five PPP misclassifications arose exclusively from false-positive or false-negative NL calls, with no errors attributable to incorrect CL matching. This reflects the nature of PPP’s decision rule: even if a CL is imperfectly matched, progression is only triggered when that lesion exhibits a ≥ 30% volume increase. In contrast, the appearance of even a single spurious NL immediately classifies the patient as progressive, making NL detection errors much more influential on PPP outcomes. The same logic extends to the TLW misclassifications, although one case was due to a CL-mismatch error. Interestingly, the algorithmic-TLW progressive category (defined as HVP + LVP) was prognostic of OS (HR: 2.285, 95% CI 1.219- 4.284, *p* = 0.010). This finding may serve as a preliminary external validation of the framework outside the [^1^⁷⁷Lu]Lu-PSMA setting [9], suggesting that its response criteria may be generalisable to less advanced disease cohorts and applicable across a broader range of treatment contexts. Taken together, these findings show that the algorithm-derived classifications preserved prognostic relevance for overall survival across all three evaluated response frameworks, consistent with the study’s final objective.

Several methodological limitations should be acknowledged. First, the accuracy of any registration-based tracking framework is inherently constrained by the quality of image registration itself. Indeed, in the present study, mismatches were strongly associated with poorer registration metrics, a finding consistent with Santoro-Fernandes et al. [15, 17]. These lesion-level errors were subsequently propagated to the patient-level classifications, as the latter misclassifications were associated with poorer NCC and higher MSE values. Second, the current implementation did not explicitly model lesion-splitting or merging events between time points—a phenomenon increasingly considered in lesion tracking algorithms. Incorporating clustering-based schemes to inform lesion correspondence would increase the robustness of true lesion identification, especially in more heterogeneous disease, and should be considered in future works. Thirdly, across all cost functions tested for one-to-one lesion pair optimisation, the matching accuracy seemed to cap around 95%. Part of this limit likely stems from the imperfection of reference annotations as these are themselves subject to inter-observer variability. For example, Huff et al. reported inter-observer disagreement in 67% of high-burden cases (> 30 lesions) and an F1-score of 0.89 between reader consensus and a similar automated lesion-tracking pipeline [10]. It should also be noted that the highest-performing cost function in the present work was identified using the same cohort on which final performance was reported, and independent validation will be necessary to establish the robustness and generalisability of this selection. Finally, the lesion segmentations were derived using a semi-automated approach, requiring expert input and introducing potential observer-related variability. Integration of AI-based segmentation methods with the automated lesion-matching framework presented here could enable fully automated patient-level response assessment in future implementations.

Overall, the algorithm achieved high matching accuracy across patients with varying lesion counts, and tracking performance appeared independent of total lesion burden and size. However, lesion burden in this cohort was generally low (media*n* = 2 lesions at both baseline and follow-up; IQR 1–4), and overall disease volumes were also relatively small (mean 7.04 mL at baseline and 6.91 mL at follow-up); thus, this apparent burden- and size-independence should be interpreted within the context of a low-volume population. As such, the findings cannot be directly extrapolated to patients with substantially higher metastatic burden without further validation. This consideration is particularly important given that the RECIP 1.0 and TLW frameworks were originally developed for metastatic settings beyond biochemical recurrence.

## Conclusion

Automated, registration-based lesion tracking can reliably reproduce expert lesion correspondences on [^68^Ga]Ga-PSMA-11 PET/CT and generate accurate, prognostically meaningful classifications across multiple PSMA-specific response frameworks. By integrating a fractional-overlap cost formulation and adaptive lesion-specific dilation, the algorithm maintained high matching accuracy and preserved overall survival associations under RECIP 1.0, PPP, and TLW. These findings highlight the feasibility of integrating fully automated lesion-tracking outputs into standardised response evaluation criteria on PSMA PET/CT imaging in a low-volume BCR setting.

## Supplementary Information

Below is the link to the electronic supplementary material.

Supplementary file1 (DOCX 322 KB)

## Data Availability

The datasets analysed during the current study are not publicly available due to patient privacy restrictions.
