# Supplementary material for: Automated lesion tracking and treatment response classification on [68Ga]Ga-PSMA-11 PET/CT across PPP, RECIP 1.0 and TLW frameworks
Source: Eur J Nucl Med Mol Imaging. 2026 Jul 16;53(12):7109–20. doi: 10.1007/s00259-026-08071-2 (PMC13633376; doi:10.1007/s00259-026-08071-2)
Supplement: Supplementary file 1 [file 259_2026_8071_MOESM1_ESM.docx]

Automated lesion tracking and treatment response classification on [^68^Ga]Ga-PSMA-11 PET/CT across PPP, RECIP 1.0 and TLW frameworks

Theo Lucas ^1,2^*, Mikaela Dell’Oro^3^, Jeremy S.L. Ong^4^, Martin A. Ebert^1,2,5^, Kaylee Molin^1,2^_,_ Jake Kendrick^1,2^

^1^School of Physics, Mathematics and Computing, University of Western Australia, Perth, Western Australia, Australia

^2^Centre for Advanced Technologies in Cancer Research, Perth, Western Australia, Australia.

^3^ Curtin Medical School, Curtin University, Perth, Western Australia, Australia

^4^Department of Nuclear Medicine, Fiona Stanley Hospital, Murdoch, Western Australia, Australia

^5^Department of Radiation Oncology, Sir Charles Gairdner Hospital, Perth, Western Australia, Australia

*Corresponding author. E-mail: [22965157@student.uwa.edu.au](mailto:22965157@student.uwa.edu.au)

# Supplementary Data

***Supplementary Figure 1. Sensitivity of lesion-matching accuracy to dilation radius.*** *Lesion-matching accuracy is shown as a function of fixed dilation radius (0–18 mm, in 3-mm increments) using the fractional-overlap cost function. The performance of the adaptive dilation strategy is also indicated. All results are reported as correct matching decisions over total matching decisions (n = 695). The figure demonstrates that moderate dilation radii yield optimal performance, while excessive dilation reduces accuracy, motivating the use of an adaptive lesion-specific dilation approach in the main analysis.*

***Supplementary Table 1. Definitions of lesion-assignment cost function variants evaluated in this study.*** *Each variant defines the cost* $C_{i,j}$ *used in the Hungarian assignment algorithm to quantify dissimilarity between baseline and follow-up lesions. Formulations include overlap-based, distance-based, and hybrid approaches. The fractional-overlap (FO) formulation, which normalises voxel intersection by the smaller lesion volume, was selected for the primary analysis due to its improved robustness to lesion size asymmetry.*

***Supplementary Table 2.*** *Matching accuracy across 4 different variants, as defined in Table 1. The accuracy for Variant B was achieved using w_D_=0.3 and w_O_ =0.7.*

| **Variant** | **Correct matching decisions/ total matching decisions** | **Accuracy (%)** |
| --- | --- | --- |
| A | 654/695 | 94.1 |
| A’ | 661/695 | 95.1 |
| B | 660/695 | 95.0 |
| C | 644/695 | 92.7 |
